# KRT18 is correlated with the malignant status and acts as an oncogene in colorectal cancer

**DOI:** 10.1042/BSR20190884

**Published:** 2019-08-13

**Authors:** Jingfeng Zhang, Sifeng Hu, Yansen Li

**Affiliations:** 1 Department of Anorectal Surgery, The Affiliated Hospital of Jining Medical University, Jining 272029, Shandong, China; 2 Department of General Surgery, Zhoucheng People’s Hospital, Zhoucheng 27350012, Shandong, China

**Keywords:** biomarker, colorectal cancer, KRT18, prognosis

## Abstract

Keratin 18 (KRT18) has been suggested to be overexpressed in most types of human tumor, but the expression pattern of KRT18 in colorectal cancer (CRC) remained unknown. In our research, KRT18 protein expression was markedly increased in CRC cancer tissues and cell lines compared with adjacent normal colorectal tissues and normal colonic epithelial cell line, respectively. Meanwhile, we observed high KRT18 expression was associated with advanced clinical stage, deep tumor invasion, lymph node metastasis, distant metastasis, poor differentiation and unfavorable prognosis in CRC patients. Multivariate Cox regression analysis showed high expression of KRT18 was an unfavorable independent predictor for overall survival in CRC patients. The *in vitro* studies indicated down-regulation of KRT18 expression depressed CRC cell viability, migration and invasion. In conclusion, KRT18 serves as an oncogenic role in CRC progression and may be a therapeutic target for promoting CRC patients’ prognosis.

## Introduction

Colorectal cancer (CRC) is the second common cancer and the second leading cause of cancer-related deaths worldwide accounting for 1800977 newly diagnosed cases and 861663 deaths in 2018 [1]. Based on 2015 Cancer Statistics in China, CRC had a remarkable upward trend in age-standardized incidence rate, and became the fifth leading cause of cancer-related deaths in China with approximately 191000 deaths in 2015 [2]. CRC is significantly threatening public health in China [3]. Despite the significant improvements in molecular targeting therapy and immunotherapy, the 5-year survival rate for CRC patients with distant metastasis or recurrence remains dissatisfactory [4–7]. Therefore, it is urgently needed to consecutively elucidate the underlying mechanism and identify novel therapeutic targets for improving the clinical outcome of CRC patients.

Keratin 18 (KRT18) is a member of the intermediate filament family of cytoskeletal protein, which is essential for tissue integrity [8]. Originally, KRT18 expression was suggested in epithelial and endothelial cells from the respiratory and gastrointestinal tract [9]. Subsequently, KRT18 was found to be aberrantly expressed in various human malignancies and correlated with clinical progression and prognosis [10,11]. However, the clinical significance and biological function of KRT18 was seldom reported in CRC. Only one study showed that normal colon tissues exhibited higher KRT18 expression than colon adenomatous polyp or carcinoma tissues [12]. In our study, we first observed the *KRT18* expression in CRC tissues and normal tissues at The Cancer Genome Atlas (TCGA) and The Genotype-Tissue Expression (GTEx) databases [13], and found that *KRT18* expression was significantly increased in colon cancer tissues and rectal cancer compared with corresponding normal tissues. Then, high expression of KRT18 was confirmed in CRC tissues and cell lines in our study, and found to be associated with malignant status in CRC patients. Survival analyses indicated that CRC patients with high KRT18 expression had shorter overall survival time than those with low KRT18 expression, and high KRT18 expression acted as an independent prognostic predictor for overall survival in CRC patients. Finally, the *in vitro* experiments revealed inhibition of KRT18 expression depressed CRC cell viability, migration and invasion.

## Materials and methods

### Database analysis

TCGA database (275 colon cancer tissues and 41 normal tissues; 92 rectal cancer tissues and 10 normal tissues) and GTEx database (308 normal tissues) were used for data analysis. In addition, analysis of TCGA and GTEx databases was performed at the GEPIA (Gene Expression Profiling Interactive Analysis, http://gepia.cancer-pku.cn/) platform.

### Clinical tissue specimens

One hundred and twelve patients with CRC in the present study received surgery or biopsy in The Affiliated Hospital of Jining Medical University or Zhoucheng People’s Hospital. The following samples were obtained and paraffin-embedded: adjacent normal colorectal tissues (*n*=36) and CRC tissues (*n*=108), which were applied to immunohistochemistry. None of the CRC cases had received anti-tumor treatment prior to pathologic diagnosis. The adjacent normal colorectal tissues were acquired from 5 cm over the margin of tumor tissues. Histopathologic examination of each tissue was verified by two pathologists. The clinical stage was determined based on 7th edition the American Joint Committee on Cancer (AJCC) TNM staging system.

### Immunohistochemistry

The 4-μm-thick CRC sections were subjected to routine deparaffinization and rehydration. Antigen retrieval was accomplished by incubating the sections in 10 mM sodium citrate buffer for 10 min and microwaving the sections for 20 min. Then, the sections were treated with 0.3% H_2_O_2_ for inhibition of endogenous peroxidase activity, and incubated with 5% nonfat dried milk for blocking of nonspecific binding. After washing, the sections were incubated with anti-KRT18 antibody (1:250 dilution, catalog ab133263, Abcam, U.S.A.) at 4°C overnight, and biotinylated secondary antibody (1:2000 dilution, Beyotime, China) for 90 min. Phosphate buffered saline was used instead of a primary antibody as a negative control. Subsequently, the sections were incubated with ABC solution for 30 min, and treated with 3,3′-diaminobenzidine (DAB) for 5 min. After Hematoxylin counterstain, the sections were dehydrated and sealed.

The KRT18 staining was scored by combining the percentage of positive tumor cells and the intensity of the staining. The percentage of positive tumor cells was scored as 0 (no staining), 1 (<10%), 2 (10–50%), 3 (50–80%) and 4 (>80%). The staining intensity was scored as 0 (no staining), 1 (weak), 2 (moderate), and 3 (strong). The final score was obtained by multiplying the score of staining intensity and the score of positive tumor cells. The CRC cases were divided into two groups: low KRT18 expression group (score 0–6) and high KRT18 expression group (score 8–12).

### Cell lines

The human normal colonic epithelial cell line (NCM460) and four human CRC cell lines (HT29, HCT116, SW480, SW620) were purchased from Cell Bank of the Chinese Academy of Sciences (Shanghai, China) and cultured in Roswell Park Memorial Institute (RPMI)-1640 medium (Gibco, U.S.A.) supplemented with 10% fetal bovine serum (FBS; Gibco, U.S.A.) at an incubator with an atmosphere of 5% CO_2_ and 37°C.

### Cell transfection

The siRNA for KRT18 (si-KRT18) and negative control (si-NC) were designed and synthesized by Sigma–Aldrich (St. Louis, MO, U.S.A.). The si-KRT18 or si-NC was transfected into CRC cells by using Lipofectamine RNAiMAX (Invitrogen, U.S.A.) according to the protocol of the manufacturer. The depletion of KRT18 by siRNA treatment was confirmed by Western blot after transfection with 72 h. The CRC cells were collected after 48 h transfection for the following assays *in vitro*.

### Cell counting kit-8 assay

CRC cells (2 × 10^3^ cells per well) were seeded in 96-well plates, and cultured for 24, 72, and 120 h. Then, cell counting kit-8 (CCK8) solutions were added in each well and incubated for 2 h at 37°C. The optical density (OD) of each was detected via a microplate reader at 450 nm. Each experiment was performed in triplicate.

### Cell migration and invasion assays

Cell migration and invasion assays were performed by trawnswell chamber (8-μm pore size, Corning, U.S.A.). For cell invasion assay, the transwell chamber was coated with Matrigel (BD Biosciences, U.S.A.). CRC cells (1 × 10^5^ cells/well) were resuspended in serum-free RPMI-1640 medium, and seeded in the upper well of the transwell chamber. The lower well of the transwell chamber was filled with RPMI-1640 medium containing 20% FBS. After culturing for 24 h, the filter inserts were removed from the chambers, and fixed with methanol and stained with Crystal Violet. Finally, the number of cells was counted in five random fields under a microscope. All assays were independently repeated at least three times.

### Western blot

CRC cells were crushed in lysis buffer (Beyotime, China) according to the protocol of the manufacturer. Equal amount of proteins were verified by BCA protein assay kit (Beyotime, China), separated by 10% sodium dodecyl sulfate/polyacrylamide gel and electroblotted on to polyvinyl difluoride membranes (Millipore, U.S.A.). Then, the membrane was immunoblotted overnight at 4°C with primary antibody against human KRT18 (1:100 dilution; catalog ab133263, Abcam, U.S.A.), and incubated with 1:3000 horseradish peroxidase–conjugated secondary antibody (Beyotime, China). The enhanced chemiluminescence system (Beyotime, China) was used to detect the signal. The bands were obtained and quantified by Quantity One (Bio-Rad, U.S.A.). Each experiment was performed in triplicate.

### Statistical analysis

All data are presented as the mean ± SD from at least three independent experiments. SPSS version 22.0 software (IBM Corporation, NY, U.S.A.) was used to analyze all results. The difference between two groups was assessed by the independent two-tailed Student’s *t* test. Chi-square test was used to estimate correlations between KRT18 expression and clinicopathological characteristics of CRC patients. Kaplan–Meier method and log-rank test were used to evaluate the overall survival difference between high KRT18 expression group and low KRT18 expression group. Univariate and multivariate Cox proportional hazards regression models were used to assess potential factors for predicting overall survival in CRC patients. A value of *P*<0.05 was considered as statistically significant.

## Results

### KRT18 expression is increased in CRC tissues and cell lines

**Figure 1:**
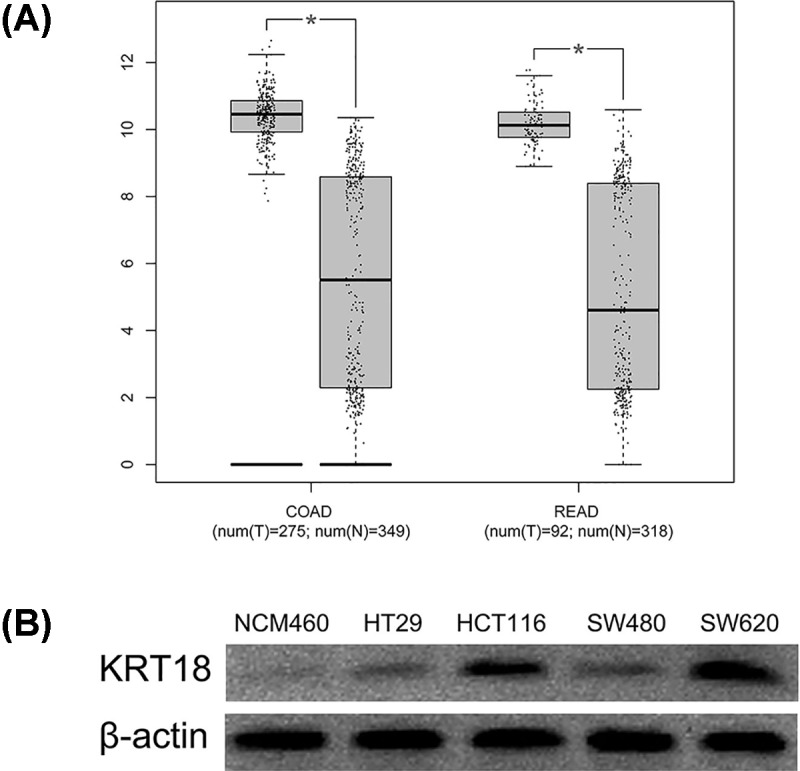
KRT18 expression is increased in CRC tissues and cell lines (**A**) Levels of KRT18 expression were dramatically increased in colon cancer and rectal cancer tissues compared with corresponding normal tissues. (**B**) KRT18 was observed in all human CRC cell lines compared with human normal colonic epithelial cell line. *P<0.001

**Figure 2:**
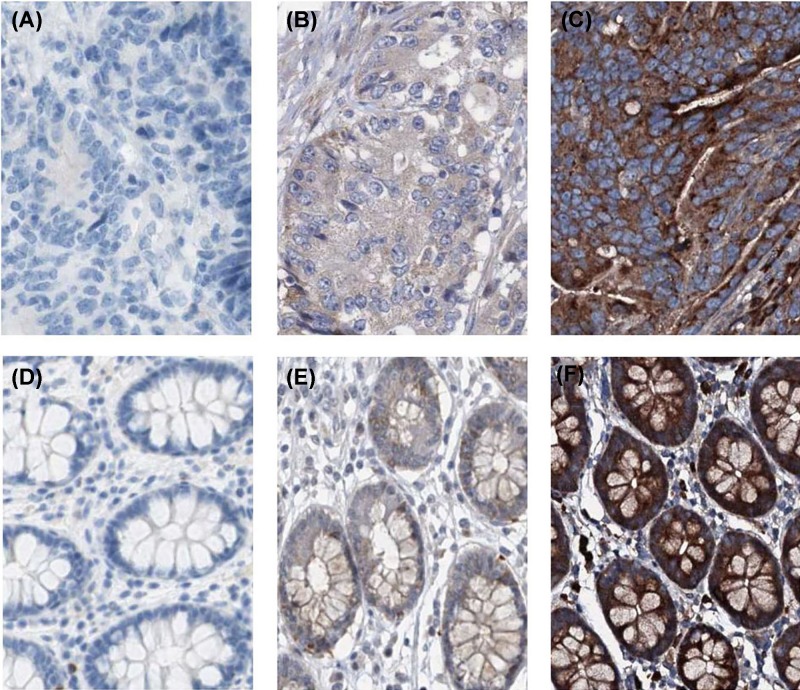
The immunohistochemical KRT18 staining in CRC tissues (**A**) Negative KRT18 expression in CRC tissues. (**B**) Low cytoplasmic KRT18 expression in CRC tissues. (**C**) High cytoplasmic KRT18 expression in CRC tissues. (**D**) Negative KRT18 expression in normal colorectal tissues. (**E**) Low cytoplasmic KRT18 expression in normal colorectal tissues. (**F**) High cytoplasmic KRT18 expression in normal colorectal tissues.

For exploring the expression pattern of KRT18 in CRC, we first observed *KRT18* expression in TCGA and GTEx databases, and found levels of KRT18 expression were dramatically increased in colon cancer and rectal cancer tissues compared with corresponding normal tissues (both *P*<0.001, Figure 1A). Furthermore, we conducted immunohistochemistry to detect the KRT18 expression in CRC tissues and normal colorectal tissues (Figure 2A–F). KRT18 showed high expression in the cytoplasm of cells. The results of immunohistochemistry showed high KRT18 staining was observed in 62 of 108 CRC tissues (57.4%) and 10 of 36 normal colorectal tissues (27.8%). The statistical analysis indicated that there was significant difference in KRT18 staining between CRC tissues and normal colorectal tissues (*P*=0.002, Table 1), which was similar to the result of TCGA database. Moreover, the expression of KRT18 was also measured in the human normal colonic epithelial cell line (NCM460) and four human CRC cell lines (HT29, HCT116, SW480, SW620) through Western blot. The result of Western blot revealed that overexpression of KRT18 was observed in all human CRC cell lines compared with human normal colonic epithelial cell line (Figure 1B).

**Table 1:** KRT18 protein expression between CRC tissues and adjacent normal tissues

Group	Cases	High KRT18 expression	Low KRT18 expression	***P***
Tumor tissues	108	62	46	0.002
Normal tissues	36	10	26	

### KRT18 expression is related with clinical progression in CRC patients

For exploring the clinical significance of KRT18 expression in CRC patients, the relationship between KRT18 expression and clinicopathological characteristics were estimated by Chi-square test. As shown in Table 2, high KRT18 expression associated with clinical stage (*P*=0.003, Table 2), tumor invasion depth (*P*=0.009, Table 2), lymph node metastasis (*P*=0.008, Table 2), distant metastasis (*P*=0.001, Table 2), and degree of differentiation (*P*=0.005, Table 2), but had no significant association with gender (*P*=0.082, Table 2), age (*P*=0.862, Table 2), family history (*P*=0.447, Table 2), and location (*P*=0.083, Table 2).

**Table 2:** Correlations between KRT18 expression and clinicopathological characteristics in CRC patients

Characteristics	*n*	High KRT18 expression	Low KRT18 expression	*P*
Age (years)				
<50	48	28	20	0.862
≥50	60	34	26	
Gender				
Female	37	17	20	0.082
Male	71	45	26	
Clinical stage				
I–II	33	12	21	0.003
III–IV	75	50	25	
Tumor invasion depth				
T1–T2	50	22	28	0.009
T3–T4	58	40	18	
Lymph node metastasis				
N0–N1	43	18	25	0.008
N2	65	44	21	
Distant metastasis				
M0	96	50	46	0.001
M1	12	12	0	
Degree of differentiation				
High or Middle	68	32	36	0.005
Low	40	30	10	
Family history				
No	88	49	39	0.447
Yes	20	13	7	
Location				
Colon	62	40	22	0.083
Rectum	46	22	24	

### KRT18 expression is related with prognosis in CRC patients

**Figure 3:**
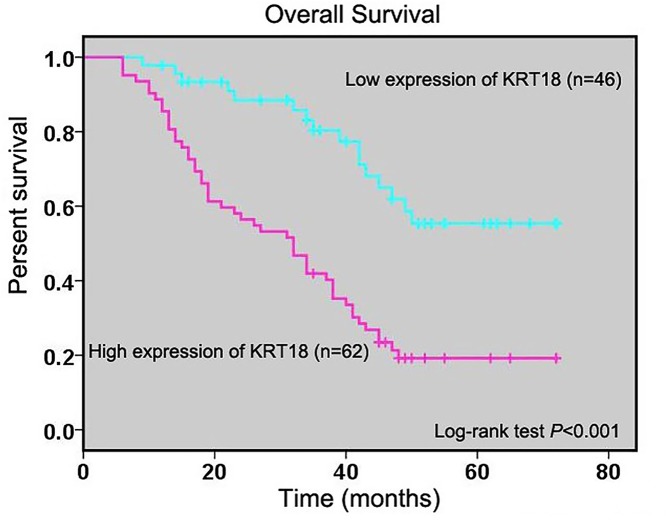
KRT18 expression is related with prognosis in CRC patients Kaplan–Meier method and log-rank test were analyzed the relationship between KRT18 expression and overall survival time of CRC patients.

To explore the influence of KRT18 expression on CRC patients’ overall survival, we analyzed the relationship between KRT18 expression and overall survival time of CRC patients. Kaplan–Meier method and log-rank test showed KRT18 expression was negatively related to overall survival time of CRC patients (*P*<0.001, Figure 3). Moreover, univariate Cox regression analysis revealed that clinical stage (*P*=0.004, Table 3), tumor invasion depth (*P*=0.022, Table 3), lymph node metastasis (*P*=0.009, Table 3), distant metastasis (*P*<0.001, Table 3), family history (*P*=0.042, Table 3), and KRT18 expression (I–IIA vs. IIB–IV, *P*<0.001, Table 3) were prognostic factors for CRC patients. Furthermore, high expression of KRT18 was found to be an unfavorable independent predictor of overall survival in CRC patients at multivariate Cox regression analysis (*P*=0.010, Table 3).

**Table 3:** Univariate and multivariate cox regression of prognostic factors for overall survival in CRC patients

Parameter	Univariate analysis			Multivariate analysis		
	HR	95% CI	*P*	HR	95% CI	*P*
Age (years)						
(<50 vs. ≥50)	0.592	0.363–0.965	0.035	0.688	0.415–1.142	0.148
Gender						
(Female vs. Male)	1.687	0.979–2.908	0.060	1.332	0.726–2.446	0.354
Clinical stage						
(I–II vs. III–IV)	2.403	1.318–4.382	0.004	1.320	0.488–3.570	0.585
Tumor invasion depth						
(T1–T2 vs. T3–T4)	1.803	1.088–2.989	0.022	1.205	0.677–2.143	0.527
Lymph node metastasis						
(N0–N1 vs. N2)	2.015	1.188–3.416	0.009	1.243	0.502–3.077	0.638
Distant metastasis						
(M0 vs. M1)	4.010	2.031–7.917	<0.001	1.871	0.850–4.121	0.120
Degree of differentiation						
(High or Middle vs. Low)	1.388	0.846–2.277	0.194	0.829	0.445–1.543	0.554
Family history						
(No vs. Yes)	1.829	1.024–3.270	0.042	1.654	0.814–3.363	0.164
Location						
(Colon vs. Rectum)	1.002	0.607–1.653	0.994	0.964	0.524–1.774	0.906
KRT18 expression						
(Low vs. High)	3.315	1.877–5.854	<0.001	2.377	1.230–4.597	0.010

Abbreviations: HR, hazard ratio; 95% CI, 95% confidence interval.

### Silencing of KRT18 expression inhibits CRC cell viability, migration, and invasion

**Figure 4:**
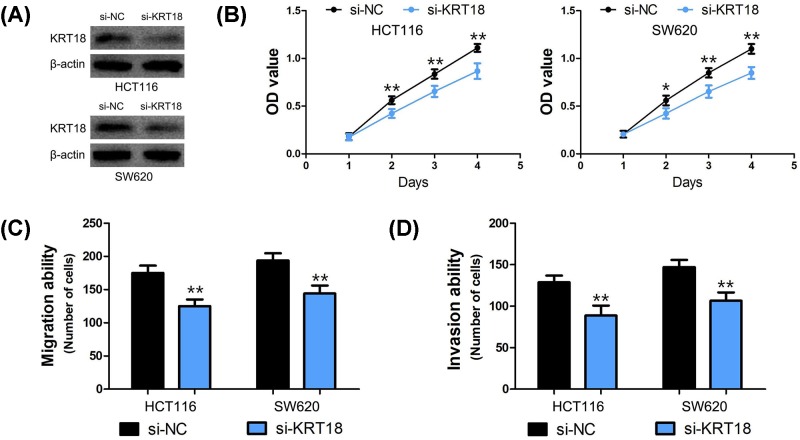
Silencing of KRT18 expression inhibits CRC cell proliferation, migration and invasion (**A**) The depletion of KRT18 by siRNA treatment was confirmed by Western blot in HCT116 and SW620 cells. (**B**) The proliferation of HCT116 and SW620 cells was strikingly suppressed after KRT18 silencing. (**C,D**) The migratory and invasive abilities of HCT116 and SW620 cells were dramatically inhibited by KRT18 knockdown. (*: *P*<0.01, **: *P*<0.001)

To explore the biological role of KRT18 in CRC cells, we performed loss-of-function studies through si-KRT18 in HCT116 and SW620 cells (Figure 4A). The CCK8 assay suggested that the viability of HCT116 and SW620 cells was suppressed after KRT18 silencing (*P*<0.01, Figure 4B). Moreover, the results of cell migration and invasion assays demonstrated that the migratory and invasive abilities of HCT116 and SW620 cells were inhibited by KRT18 knockdown (*P*<0.001, Figure 4C,D).

## Discussion

KRT18 is a type I intermediate filament protein that extends from the surface of the nucleus to the cell membrane, and involved in a variety of cellular processes including cell proliferation, cell cycle, apoptosis, motility, and cell signaling [14]. In recent decade, KRT18 was suggested to be up-regulated in various human tumor tissues including lung cancer [15], invasive breast cancer [16], esophageal cancer [17], gastric cancer [18], hepatocellular carcinoma [19], bladder cancer [20], ovarian cancer [21], oral squamous cell carcinoma [22], pancreatic cancer [23], prostate cancer [24], and so on. Drew et al. [12]performed multivariate discriminant analysis on the hCellMarkerPlex gene expression data including human colon normal tissues, colon adenomatous polyp tissues and colon carcinoma tissues, and found that colon normal tissues had significantly low *KRT18* expression compared with colon adenomatous polyp tissues and colon carcinoma tissues. In our study, we further found that KRT18 protein expression was increased in CRC cancer tissues and cell lines. Generally, KRT18 is overexpressed in most types of human cancer.

We further investigated the clinical significance of KRT18, and observed high KRT18 expression was positively associated with clinical stage, tumor invasion depth, lymph node metastasis, distant metastasis, and degree of differentiation. In lung cancer patients, KRT18 expression was suggested to be correlated with clinical lymph node metastasis [25]. In addition, KRT18 expression had 4.6-times increase in high metastatic lung cancer cell line compared with low metastatic lung cancer cell line [26]. In hepatocellular carcinoma patients, levels of KRT18 expression were positively correlated with lymph node metastasis and aggressive phenotype [27,28]. Moreover, Afrem et al. [29] reported KRT18 overexpression was correlated with advanced clinical stage, poor differentiation, and high invasion pattern in oral squamous cell carcinoma patients. In bladder cancer, Wild et al. [20] and Catto et al. [30] conformably found KRT18 served as one of artificial intelligence-selected genes for predicting clinical progression. Moreover, Yin et al. [31] suggested high expression of KRT18 was associated with tumor aggressiveness and paclitaxel resistance in prostate cancer patients. In breast cancer, Chalabi et al. [32] revealed that high expression of KRT18 often observed in luminal B patients from Lebanon, Tunisia, and Morocco, and in luminal A patients from France. Besides, Kilic-Baygutalp et al. [17] found KRT18 expression was positively associated with clinical stage, tumor stage, and metastasis stage in patients with esophageal cancer. In gastric cancer, high KRT18 expression was suggested to be associated with positive lymph nodes, advanced clinical stage, and chemoresistance [33,34].

The prognostic significance of KRT18 is still not well understood in human tumors. Zhang et al. [35] reported that KRT18 overexpression was correlated with short overall survival and disease-free survival in lung cancer patients. In hepatocellular carcinoma patients, KRT18 overexpression served as an unfavorable prognostic predictor for 1-year survival [36]. Moreover, Yang et al. [37] found positive KRT8/18 was an independent prognostic factor for overall survival in patients with esophageal squamous cell carcinoma. In nasopharyngeal carcinoma patients, Huang et al. [38] showed high expression of KRT18 had high specificity for predicting poor prognosis. However, Morisaki et al. [39] indicated that KRT18 expression had no relationship with overall survival time in gastric cancer patients. There was no report about the prognostic significance of KRT18 in CRC patients. In our study, we also found KRT18 overexpression acted as an unfavorable independent predictor of overall survival in CRC patients.

In past decades, KRT18 has been found to function as oncogenic lncRNA in several types of human malignancy. In lung cancer cells, Zhang et al. [35] showed down-regulation of KRT18 that inhibited cell migration and elevated the sensitivity to paclitaxel. In addition, down-regulation of KRT18 was found to depress epithelial cancer cell motility, invasion, and cisplatin sensitivity [11]. In hepatocellular carcinoma, KRT18 deficiency accelerated liver tumor development [40]. In CRC cells, we observed silencing of KRT18 expression inhibited CRC cell viability, migration, and invasion. In colonic epithelial cells, Lähdeniemi et al. [41] found KRT18 interacted with Notch1 and regulate Notch1 signalling activity. Unfortunately, we did not further explore the molecular mechanism of KRT18 in CRC due to limited research fund. However, Zhang et al. [25] reported that KRT18 expression was directly regulated by EGR1, which has been showed to function as a tumor suppressor in lung cancer. Moreover, Fortier et al. [11] found knockdown of KRT18 led to PI3K/Akt/NF-κB hyperactivation and elevated MMP2 and MMP9 expression, but no had effects on epithelial–mesenchymal transition markers. Meanwhile, knockdown of KRT18 was elevated Fas receptor membrane targeting in a claudin1-dependent manner, thus enhancing the activation of the extrinsic apoptosis pathway [11]. Therefore, more studies would be needed to explore and verify the role of KRT18 in tumorigenesis.

In conclusion, KRT18 is overexpressed in CRC tissues and cell lines, and associated with tumor progression and overall survival. Down-regulation of KRT18 expression depresses CRC cell viability, migration, and invasion *in vitro*.

## Abbreviations

CCK8: cell counting kit-8
CRC: colorectal cancer
FBS: fetal bovine serum
GTEx: The Genotype-Tissue Expression
KRT18: Keratin 18
MMP: matrix metalloprotein
RPMI: Roswell Park Memorial Institute
TCGA: The Cancer Genome Atlas
